# The obesity paradox in peritoneal dialysis: reassessing optimal BMI via longitudinal time in range analysis in a single-center cohort

**DOI:** 10.3389/fnut.2026.1982039

**Published:** 2026-10-22

**Authors:** Meihua Wang, Xiaohong Zeng, Dehui Liu, Pingli Chen, Zhiqiang Peng

**Affiliations:** 1 Department of Nephrology, Ganzhou People's Hospital, Ganzhou Hospital-Nanfang Hospital, Southern Medical University, Ganzhou, China; 2 Department of Respiratory and Critical Care Medicine, Ganzhou People's Hospital, Ganzhou Hospital-Nanfang Hospital, Southern Medical University, Ganzhou, China

**Keywords:** death, obesity, peritoneal dialysis, time above target range, time in range

## Abstract

**Background:**

The optimal BMI target for mortality prevention in PD patients remains unclear, with existing evidence largely limited to single-time-point measurements that ignore cumulative exposure.

**Methods:**

This single-center retrospective cohort study enrolled 670 PD patients with repeated BMI measurements at baseline, 12 months, and 36 months. BMI time above target range (TAR) was calculated for each integer threshold from 18 to 32 kg/m^2^ using the Rosendaal linear interpolation method. Multivariable Cox proportional hazards models were used to identify the threshold with the strongest protective association with all-cause mortality. BMI time in target range (TTR) within the optimal range was then calculated, and its association with mortality was assessed using a landmark design (from 36 months onward), with adjustments for age, diabetes, serum albumin, estimated glomerular filtration rate, high-density lipoprotein cholesterol, 24-h urine volume, 3-year cumulative peritonitis episodes.

**Results:**

Over a median follow-up of 2.3 years, 75 deaths occurred. The TAR threshold scan revealed a non-linear protective pattern, with the nadir of mortality risk at 26 kg/m^2^ [HR per 10% TAR increment: 0.82, 95% CI: 0.73–0.92, *P* = 0.018 (Bonferroni-corrected)]. Notably, TTR of the 26–30 kg/m^2^ range was significantly associated with reduced mortality risk (HR per 10% increment: 0.77, 95% CI: 0.62–0.96, *P* = 0.02), whereas TTR of the 24–25.9 kg/m^2^ range did not reach statistical significance (HR = 0.98, 95% CI: 0.89–1.08, *P* = 0.77).

**Conclusions:**

Sustained BMI within 26–30 kg/m^2^ was associated with lower all-cause mortality in Chinese PD patients, suggesting a potentially higher optimal BMI target. However, these findings are exploratory and require confirmation in larger, multi-center studies.

## Introduction

Peritoneal dialysis (PD) is a key renal replacement therapy for end-stage renal disease (ESRD), accounting for 11% of global dialysis and 9% of all kidney replacement therapy (1, 2). PD use is rising in China, the US, and Thailand (1). Compared with hemodialysis, PD offers greater autonomy and flexibility, improving quality of life. Nonetheless, long-term survival remains poor, with cardiovascular disease, infections, and malnutrition-inflammation-atherosclerosis syndrome as major causes of death (3). Hence, identifying modifiable risk factors is essential for better outcomes.

Obesity and overweight are well-established risk factors for mortality in the general population. In ESRD patients, however, the “obesity paradox”-where higher body mass index (BMI) is paradoxically associated with better survival-has been well-documented in hemodialysis (4), but findings in PD populations remain inconsistent. Some studies reported protective or neutral effects of higher BMI (5–7), while others suggested increased mortality beyond thresholds such as 25.71 kg/m^2^ (8). A recent meta-analysis confirmed that underweight PD patients had significantly higher mortality than normal-weight individuals, whereas overweight showed no significant difference (9).

These conflicting findings highlight the absence of an established BMI target for PD patients, and the optimal threshold remains controversial, with studies reporting disparate cutoffs ranging from 21.4 to 35 kg/m^2^ (6, 8, 10, 11). Notably, most of these studies relied on baseline single-point BMI measurements or simple changes between two time points. However, BMI fluctuates dynamically over time, and such approaches fail to capture the cumulative burden of sustained BMI exposure. Although a few studies have employed longitudinal analyses (12–14), they primarily focused on the magnitude of BMI decline or variability, rather than the cumulative duration of exposure. To address these methodological gaps and the lack of large-scale longitudinal evidence in Chinese PD populations, we adopted a novel approach.

Inspired by the “time in range” concept in diabetes management, which has proven superior to single HbA1c measurements (15), we applied this framework to BMI and introduced two novel longitudinal metrics: BMI time above target range (TAR) and BMI time in target range (TTR). TAR quantifies the cumulative percentage of time that BMI exceeds a given threshold (16), while TTR measures the time spent within a specified optimal range.

Therefore, we conducted this retrospective single-center cohort study to (1) identify the optimal mortality-related BMI threshold using BMI TAR across thresholds from 18 to 32 kg/m^2^, and (2) validate whether maintaining BMI within the derived target range using TTR confers survival benefits in PD patients.

## Methods

### Patient population

This retrospective cohort study was conducted at a single peritoneal dialysis center in Ganzhou people's hospital. We enrolled patients aged ≥18 years who had been on continuous ambulatory peritoneal dialysis (CAPD) for 3 months and presented for their scheduled re-examination visit at that time (defined as the baseline visit) between 1 January 2014 and 31 December 2023. Patients were excluded if they had missing baseline BMI (*n* = 2), insufficient longitudinal BMI data (fewer than two BMI measurements during the first 3 years of follow-up, *n* = 287), died before the 3-year landmark (*n* = 72), transferred to hemodialysis (*n* = 75), underwent kidney transplantation (*n* = 23), or were lost to follow-up (*n* = 18), or had other reasons for exiting before the landmark (*n* = 73). Hence, the final cohort consisted of 670 participants (Figure 1). This study was approved by the Research Ethics Committee of Ganzhou people's hospital, Ganzhou, China (ethics number PJB2024-023-01).

**Figure 1:**
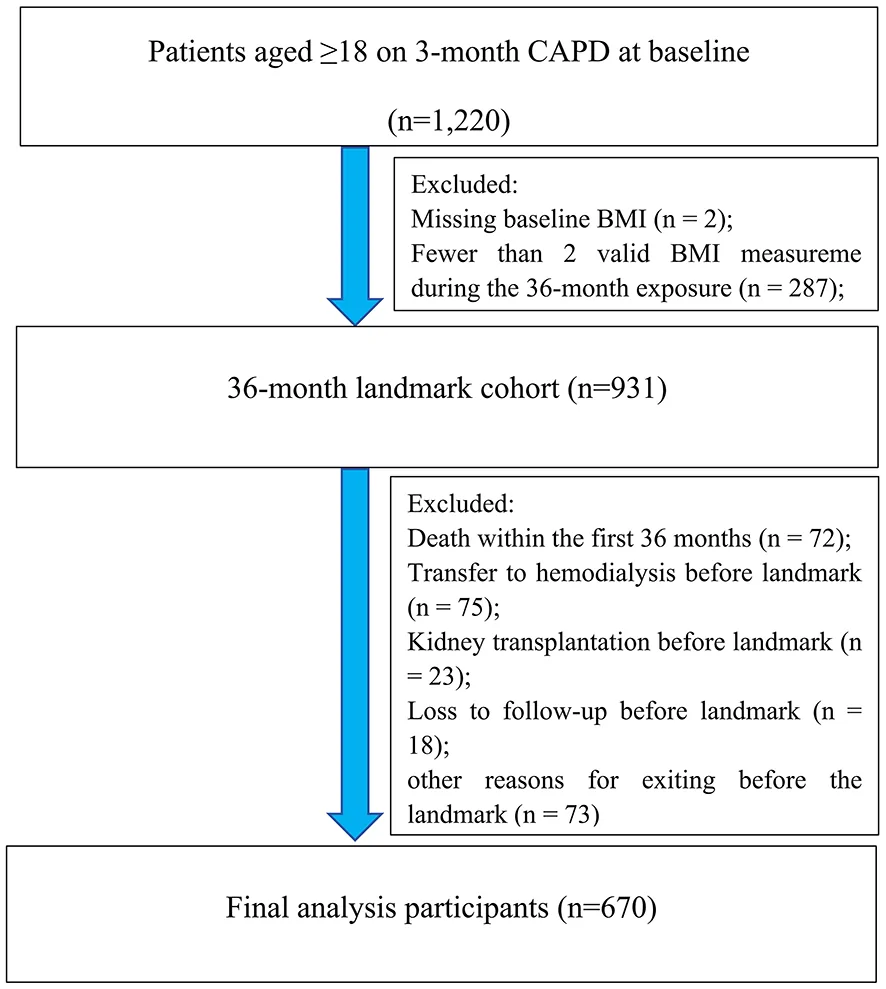
Flow chart for eligibility screening and inclusion of the study population. CAPD, continuous ambulatory peritoneal dialysis; BMI, body mass index.

### Data collection and measurements

All data were retrieved from electronic medical records in Ganzhou people's hospital. Biochemical parameters were measured using standardized and automated methods at the participating center. Baseline demographic characteristics, including age, sex, lifestyle behaviors (smoking and alcohol drinking), PD initiation data, and comorbid conditions [hypertension and diabetes mellitus], were extracted from medical records. Blood samples for biochemical assays, including serum creatinine, albumin (ALB), calcium, phosphorus, potassium, intact parathyroid hormone (iPTH), fasting glucose (Glu), serum uric acid(UA), alanine aminotransferase (ALT), aspartate aminotransferase (AST), total cholesterol (Tcho), low-density lipoprotein cholesterol (LDL-c), triglycerides (TG), high-density lipoprotein cholesterol (HDL-c), ferritinprotein, C-reactive protein (CRP) and hemoglobin (HGB), were collected at baseline (at the time of study enrollment) and 12 and 36 months of follow-up. Clinical parameters, such as systolic and diastolic blood pressure (SBP and DBP), 24-h urine volume, dialysis vintage and peritoneal equilibration test (PET) categories, were recorded at baseline. We estimated the eGFR using the 2021 CKD-EPI equation, which is based on serum creatinine measurements (17). This formula has been shown to perform well in Chinese populations (18). Serial BMI values were recorded at baseline, 12 months, and 36 months. The cumulative number of peritonitis episodes occurring during the 0–36 month exposure period was documented.

Missing data were assumed to be missing at random (MAR). We used multiple imputation by chained equations (MICE) with *m* = 20 imputed datasets (R edition 4.3 using the “mice” package). The imputation model included all variables with missing values (proportions shown in Supplementary Table S3), together with death and survival time as auxiliary variables. Convergence was assessed using trace plots, and the distributions of observed and imputed values were compared using density plots (Supplementary Figure S1). Estimates from the imputed datasets were combined using Rubin's rules.

### BMI and its longitudinal metrics

We evaluated longitudinal BMI exposure primarily using two metrics: time above target range (TAR) and time in target range (TTR). TAR was defined as the percentage of time during which BMI exceeded a given threshold (16), while TTR was defined as the percentage of time during which BMI remained within the target range. Both metrics were calculated by linear interpolation using the Rosendaal method (16, 19). To derive these indices, height and body weight were measured by trained healthcare professionals using calibrated stadiometers and digital scales, with participants wearing light clothing and no shoes, and BMI was calculated as weight (kg) divided by height squared (m^2^). Owing to the large proportion of participants who had a TTR of zero in the first 3 years, participants with a TTR of 0% were used as the reference category, and all remaining participants (those with TTR > 0%) were categorized into the exposed group in the analysis of this study. The detailed calculation process for these metrics was illustrated in Supplementary Figure S2.

### Assessment of outcomes

The primary outcome was all-cause mortality, defined as death from any cause. Deaths were ascertained through electronic medical records and complemented by telephone follow-up for patients who were lost to regular hospital visits. The follow-up for all outcomes began after the 3-year exposure period (i.e., from the 36-month landmark), consistent with the study design that used the first 3 years of BMI measurements to calculate longitudinal exposure metrics. All participants were followed from the 36-month landmark until death, loss to follow-up, or the end of the study (30 June 2026), whichever came first. The detailed calculation procedure for the follow-up duration is presented in Supplementary Figure S3.

### Statistical analysis

Continuous variables were summarized as mean (SD) or median (IQR) depending on distributional normality. Categorical variables were presented as counts and percentages. Between-group comparisons were performed using ANOVA tests or chi-square tests, accordingly.

To determine the optimal BMI threshold for mortality prediction, we conducted a threshold-scanning analysis using time-dependent Cox models. BMI TAR was calculated for each integer threshold from 18 to 32 kg/m^2^ (15 thresholds in total) and modeled as a continuous exposure. For every 10% increment in TAR, we derived the hazard ratio (HR) and 95% confidence interval (CI) for all-cause mortality separately for each threshold. To account for multiple testing, Bonferroni correction was applied across the 15 thresholds. A Bonferroni-adjusted *P*-value < 0.05 (equivalent to a raw *P*-value < 0.05/15 = 0.0033) was considered statistically significant.

Based on the threshold identified from the preliminary TAR analysis, we categorized TTR into two prespecified groups (24–25.9 kg/m^2^ vs. 26–30 kg/m^2^) and analyzed it as a continuous variable. To validate this threshold, we compared survival distributions between the two TTR groups using Kaplan–Meier curves with log-rank tests. We further evaluated the association between TTR and all-cause mortality using multivariable Cox proportional hazards models under a landmark design, with the landmark time set at 3 years after enrollment. Three sequentially adjusted models were constructed. Model 1 crude model. Model 2 further adjusted for age at recruitment (years, continuous), diabetes (yes vs. no). Model 3 additionally included eGFR (ml/min/1.73 m^2^, continuous), ALB (g/L, continuous), HDL-c (mmol/L, continuous), 24-h urine volume (ml, continuous), cumulative peritonitis episodes during the 0–36 month exposure period (counts, continuous). *Post-hoc* power was based on the observed HR for BMI-TTR of 26–30kg/m^2^ per 10% increment, 75 events, 7 covariates, and a two-sided alpha of 0.05. PET was excluded from the primary model (EPV < 10); a univariable Cox regression for high-transport status was performed using complete cases. Due to the limited number of deaths, we instead performed descriptive subgroup analyses stratified by age (< 65 vs. ≥65), diabetes status (Yes vs. No), and median-split serum albumin level (low vs. high), and exploratory interaction tests using Firth's penalized Cox regression, reporting interaction *P*-values when estimable. Several sensitivity analyses were undertaken to test the robustness of our findings: (1) repeating all analyses on the complete-case dataset without imputation; (2) recalculated TTR using a step-function interpolation method; (3) a Firth penalized Cox regression was used owing to the limited number of events; (4) calculating the *E*-value to quantify the potential impact of unmeasured confounding. Fine-Gray models explored cardiovascular and infection-related mortality, treating other deaths as competing events.

All statistical procedures were carried out using R software (version 4.5.3). A two-sided *P* < 0.05 was considered indicative of statistical significance.

## Results

### Study participants and characteristics

A total of 670 participants were included in the current study, as illustrated in the flowchart (Figure 1). Among them, the mean age was 48.2 years (SD, 12.3 years), and 54.5% were male. Based on the percentage of time during which the BMI-TTR remained within the range of 26–30kg/m^2^, patients were divided into two groups: those with 0% and those with >0% of time in this range. The baseline characteristics of the cohort were summarized in Table 1. Participants with >0% time in range seemed to have higher baseline BMI, 24-h urine volume, and baseline serum eGFR level, CRP level, TG level, and have lower baseline serum HDL-c level, and more likely to have a history of diabetes or suffering peritonitis episodes during the 0–36 month exposure period. Baseline characteristics of those excluded from the analysis were similar to those who were included in the analysis (Supplementary Table S1). Baseline characteristics stratified by outcome status (all-cause) are summarized in Supplementary Tables S2.

**Table 1:** Baseline clinical characteristics according to categories of BMI TTR of 26–30 kg/m^2^ during the first 3 years.

Characteristics	Overall, *n* (%)	BMI TTR, %		*p*-values
		Never within target (TTR = 0%)	Any time within target (TTR > 0%)	
**Total**	670	548	122	–
Demographic				
Age, mean (SD), yr.	48.2 (12.3)	48.3 (12.6)	47.8 (10.7)	0.667
Male, *n* (%)	365 (54.5)	290 (52.9)	75 (61.5)	0.106
Smoking, *n* (%)				0.621
No	583 (87.0)	479 (87.4)	104 (85.2)	
Yes	87 (13.0)	69 (12.6)	18 (14.8)	
Drinking, *n* (%)				0.153
No	617 (92.1)	509 (92.9)	108 (88.5)	
Yes	53 (7.9)	39 (7.1)	14 (11.5)	
PET, *n* (%)				0.166
Low transport	41 (6.2)	35 (6.5)	6 (5.0)	
Low average transport	270 (40.9)	214 (39.7)	56 (46.3)	
High average transport	291 (44.1)	237 (44.0)	54 (44.6)	
High transport	58 (8.8)	53 (9.8)	5 (4.1)	
Dialysis month, mean (SD), mon.	58.91 (24.82)	59.60 (24.89)	55.80 (24.37)	0.127
Laboratory test indicators, mean (SD)				
eGFR (ml/min/1.73m^2^)	6.19 (2.75)	6.07 (2.71)	6.73 (2.90)	0.017
UA (umol/L)	519.28 (148.99)	520.95 (149.48)	511.81 (147.14)	0.54
CRP (mg/L)	14.16 (23.94)	12.86 (19.44)	19.76 (37.23)	0.007
K (mmol/L)	4.62 (0.81)	4.62 (0.80)	4.64 (0.82)	0.74
P (mmol/L)	1.89 (0.59)	1.90 (0.60)	1.83 (0.55)	0.244
Ca (mmol/L)	3.60 (40.91)	3.95 (45.24)	2.04 (0.22)	0.641
iPTH (pg/L)	409.89 (338.39)	410.08 (337.00)	409.02 (345.91)	0.975
Ferroprotein (ng/ml)	251.09 (244.21)	242.60 (237.83)	288.99 (268.66)	0.059
ALT (U/L)	19.45 (17.67)	19.84 (18.48)	17.68 (13.35)	0.221
AST (U/L)	20.97 (13.73)	21.35 (14.48)	19.28 (9.58)	0.133
ALB (g/L)	36.23 (5.16)	36.23 (5.10)	36.22 (5.48)	0.995
Tcho (mmol/L)	4.34 (1.23)	4.35 (1.22)	4.30 (1.28)	0.652
Ldl -c (mmol/L)	2.49 (0.93)	2.47 (0.93)	2.58 (0.94)	0.264
TG (mmol/L)	1.60 (0.93)	1.54 (0.91)	1.88 (0.95)	< 0.001
Hdl-c (mmol/L)	1.18 (0.49)	1.21 (0.49)	1.04 (0.45)	< 0.001
Glu (mmol/L)	6.00 (9.73)	6.05 (10.74)	5.77 (1.91)	0.773
HGB (g/L)	82.02 (19.25)	81.65 (19.41)	83.70 (18.49)	0.287
Comorbidities, *n* (%)				
Hypertension	610 (91.0)	498 (90.9)	112 (91.8)	0.881
Diabetes	68 (10.1)	41 (7.5)	27 (22.1)	< 0.001
Cumulative peritonitis episodes (0–36 month exposure period)	0.48 (0.80)	0.45 (0.75)	0.63 (0.97)	0.042
Examination, mean (SD)				
24-h urine volume (ml)	959.03 (421.56)	938.96 (405.92)	1049.18 (477.16)	0.009
SBP (mmHg)	135 (17)	135 (16)	135 (18)	0.941
DBP (mmHg)	84 (11)	84 (10)	85 (13)	0.382
BMI (kg/m^2^)	21.91 (3.24)	21.07 (2.67)	25.65 (2.90)	< 0.001

PET, peritoneal equilibration test; eGFR, estimated glomerular filtration rate; UA, uric acid; CRP, C-reactive protein; K, potassium; P, phosphorus; Ca, calcium; iPTH, intact parathyroid hormone; ALB, albumin; ALT, alanine aminotransferase; AST, aspartate aminotransferase; LDL-c, low-density lipoprotein cholesterol; Tcho, total cholesterol; HDL-c, high-density lipoprotein cholesterol; TG, triglyceride; Glu, glucose; HGB, hemoglobin; SBP, systolic blood pressure. DBP, diastolic blood pressure; BMI, body mass index.

### Association between different predefined BMI TARs and mortality risk

Across 1859.34 person-years of observation (median follow-up of 2.3 years, IQR: 1.15–3.72 years), 75 deaths occurred, corresponding to a rate of 40.3 per 1,000 person-years. We evaluated the association between TARs across the range of 18–32 kg/m^2^ and all-cause mortality. Over this entire threshold spectrum, all estimated hazard ratios were below 1.0, suggesting a consistent protective tendency. Nevertheless, after Bonferroni correction, statistical significance was achieved only for thresholds between 24 and 26 kg/m^2^ (Figure 2); thresholds outside this interval (i.e., < 24 kg/m^2^ or >26 kg/m^2^) did not reach significance. Notably, the lowest HR among all cut-offs was observed at 26 kg/m^2^, indicating this threshold as the most favorable in terms of risk reduction.

**Figure 2:**
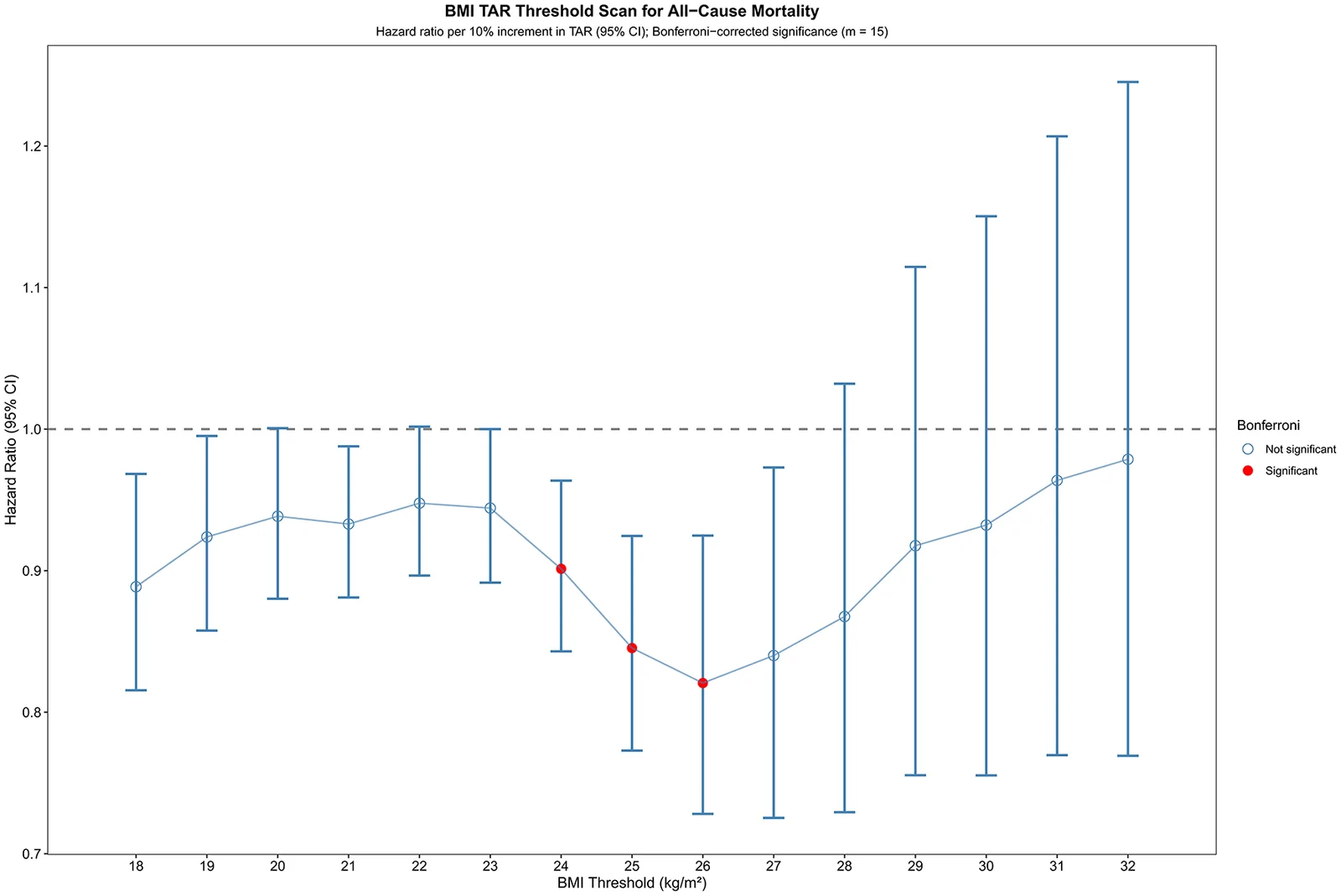
The association of TARs using different BMI thresholds with all-cause. The model was adjusted for adjusted for age, diabetes, eGFR, ALB, HDL-c, 24-h urine volume, 3-year cumulative peritonitis episodes. Data are represented as HR (95% CI) for mortality by each 10% increase in different predefined TARs. TAR, time above target range; BMI, body mass index; HR, hazard ratio; CI, confidence interval.

### Association between BMI TTR and mortality risk

Following the identification of 26 kg/m^2^ as the BMI threshold conferring the lowest mortality hazard in the TAR analysis, we extended our investigation to evaluate the prognostic role of BMI TTR using two prespecified target intervals: 24–25.9 kg/m^2^ and 26–30 kg/m^2^. The time-to-event distributions are displayed in Figure 3. Compared to the TTR = 0% group, maintaining TTR>0% within the 26–30 kg/m^2^ range was associated with a significantly lower risk of all-cause mortality. In contrast, for the 24–25.9 kg/m^2^ range, there was no significant risk reduction, suggesting a lack of additional benefit at this lower BMI threshold. Table 2 summarizes the Cox regression models used to examine the associations between TTR metrics and all-cause mortality. For the 24–25.9 kg/m^2^ range, after full adjustment for demographic, medical history, and clinical covariates, compared with the TTR of 24–25.9 kg/m^2^ = 0%, the HR for the 24–25.9 kg/m^2^ > 0% is 0.75 (95% CI: 0.46–1.23). For every 10% increment in TTR, the corresponding continuous HR was 0.98 (95% CI: 0.89–1.08). For the 26–30 kg/m^2^ range, the corresponding HR is 0.27 (95% CI: 0.11–0.68) for the categorical model, and 0.77 (95% CI: 0.62–0.96) for the continuous model. However, statistical significance was achieved exclusively for the 26–30 kg/m^2^ range, where every TTR metric (whether categorical or continuous) yielded a significant inverse association with mortality. With 75 events and 7 covariates, the study had 63% power to detect the observed effect size for BMI-TTR of 26–30 kg/m^2^ per 10% increment (HR = 0.77) at a two-sided alpha of 0.05. In contrast, none of the corresponding estimates for the 24–25.9 kg/m^2^ range reached conventional significance, although their point estimates were also less than unity. Furthermore, restricted cubic spline analysis confined to the 26–30 kg/m^2^ interval revealed a linear dose-response relationship (*p* for non-linearity = 0.21) (Figure 4), suggesting that within this range, a higher percentage of time spent in the target BMI zone is linearly associated with a lower risk of death.

**Figure 3:**
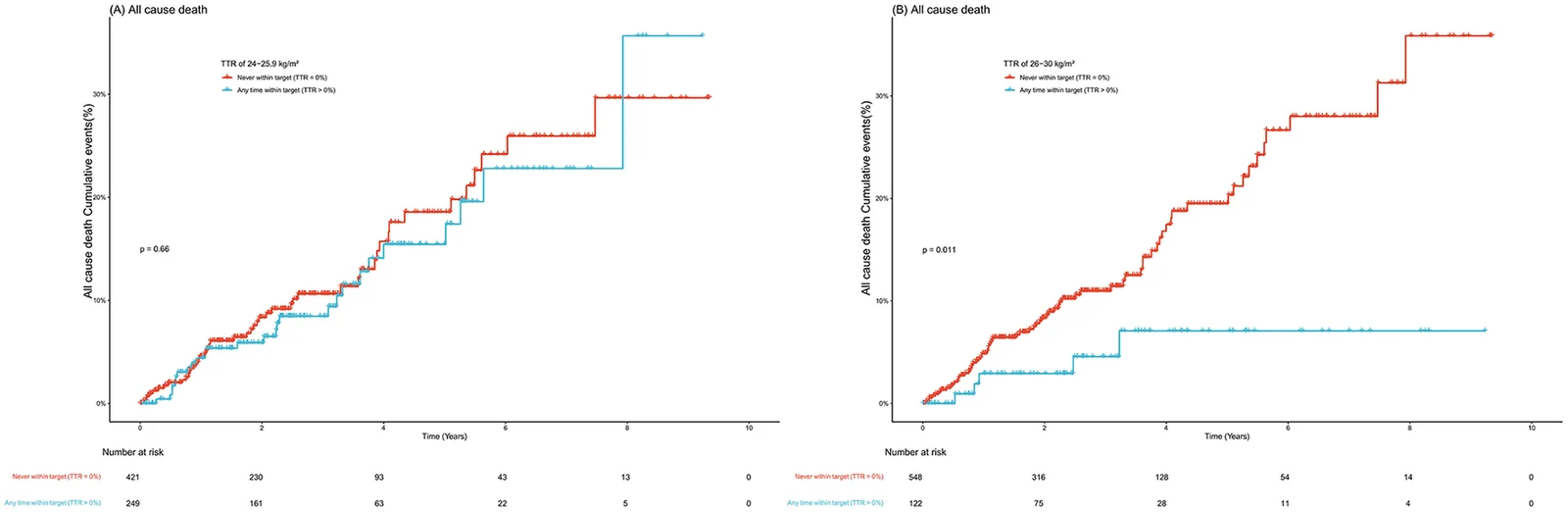
Time-to-event distribution for death, stratified by **(A)** BMI-TTR of 24–25.9 kg/m^2^ and **(B)** BMI-TTR of 26–30 kg/m^2^.

**Table 2:** Cox regression models of the association between BMI-TTR and death.

Variables	Cases/person years	Model 1		Model 2		Model 3	
		HR (95% CI)	*P*-value	HR (95% CI)	*P*-value	HR (95% CI)	*P*-value
BMI-TTR24–25.9kg/m^2^							
As category variable							
Never within target (TTR = 0%)	48/1144.56	Reference		Reference		Reference	
Any time within target (TTR > 0%)	27/714.78	0.90(0.56–1.44)	0.66	0.79(0.49–1.28)	0.34	0.75(0.46–1.23)	0.26
As continuous variable							
BMI–TTR of 24–25.9kg/m^2^ per 10% increment		0.99(0.91–1.09)	0.98	0.98(0.89–1.08)	0.76	0.98(0.89–1.08)	0.77
BMI-TTR 26–30kg/m^2^							
As category variable							
Never within target (TTR = 0%)	70/1524.00	Reference		Reference		Reference	
Any time within target (TTR > 0%)	5/335.34	0.32(0.13–0.81)	0.02	0.29(0.12–0.74)	0.01	0.27(0.11–0.68)	0.01
As continuous variable							
BMI-TTR of 26–30 kg/m^2^ per 10% increment		0.79(0.64–0.98)	0.04	0.78(0.63–0.96)	0.03	0.77(0.62–0.96)	0.02

Model 1: crude model.

Model 2: adjusted for age, diabetes.

Model 3: adjusted for adjusted for age, diabetes, eGFR, ALB, HDL-c, 24-h urine volume, cumulative peritonitis episodes during the 0–36 month exposure period.

**Figure 4:**
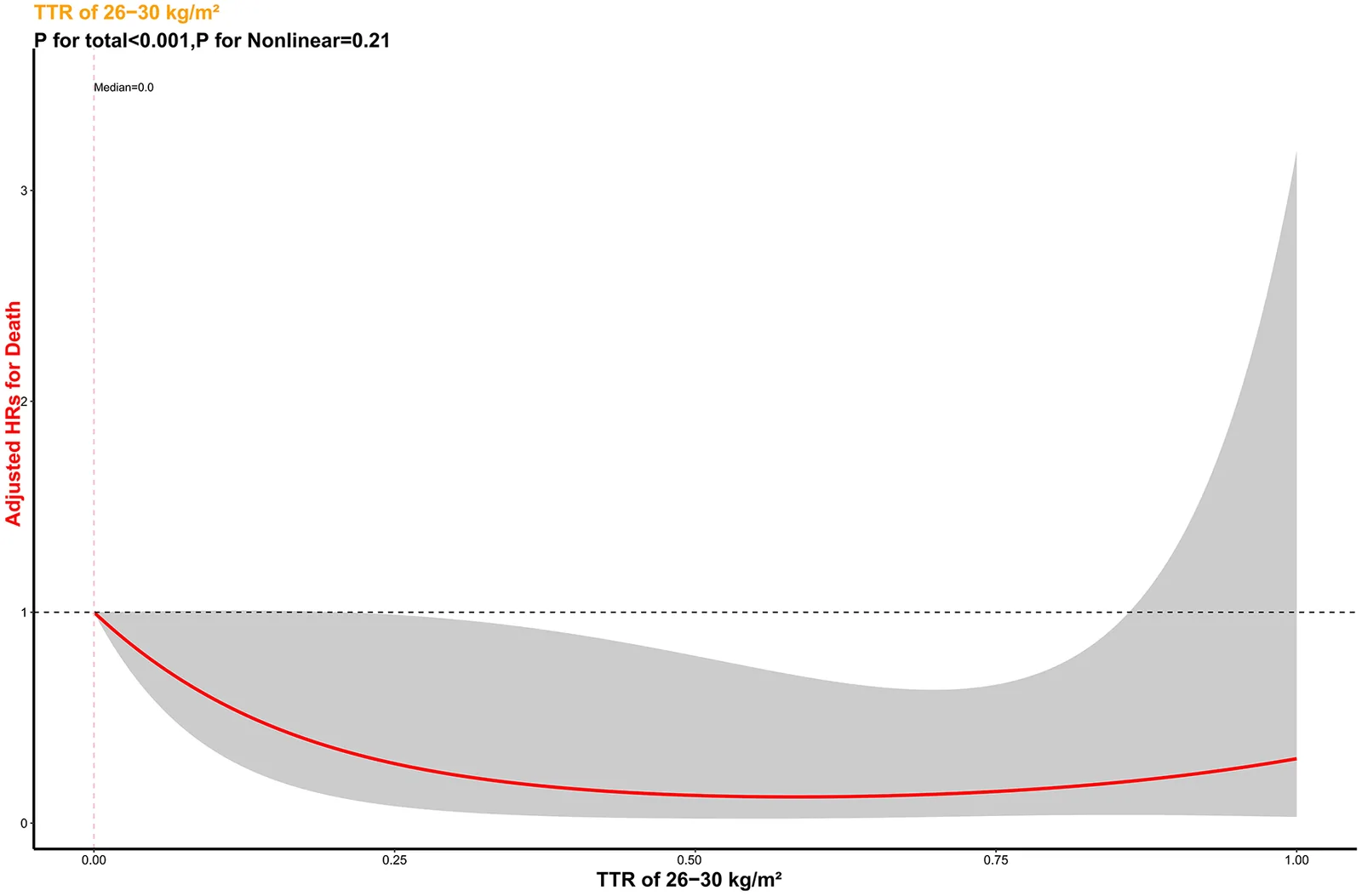
Restricted cubic spline model shows relationship of BMI-TTR of 26–30 kg/m^2^ with death. Model adjusted for adjusted for age, diabetes, eGFR, ALB, HDL-c, 24-h urine volume, cumulative peritonitis episodes during the 0–36 month exposure period.

### Sensitivity analyses

To further ensure the robustness of the present study, several sensitivity analyses were also performed. In complete-case analysis, step-function TTR, and Firth penalized Cox regression, the results were consistent with the primary analysis (Supplementary Table S4). The distribution of cause-specific deaths is presented in Supplementary Figure S4. In exploratory competing-risks analyses, each 10% increment in BMI-TTR of 26–30 kg/m^2^ was significantly associated with cardiovascular mortality (sHR = 0.68, 95% CI 0.53–0.88, *P* = 0.004), whereas each 10% increment in BMI-TTR of 26–30 kg/m^2^ was not significant associated with infection-related mortality (sHR = 0.93, 95% CI 0.71–1.21, *P* = 0.58) (Supplementary Table S5). These analyses were underpowered. The *E*-values for the association with all-cause mortality were 1.9 when TTR of 26–30 kg/m^2^ was treated as a continuous variable and 6.8 when categorized as >0 vs. 0%, suggesting that unmeasured confounding would need to be of substantial magnitude to fully explain away the observed protective associations.

### Exploratory analyses

In a univariable complete-case analysis, high-transport status was associated with all-cause mortality (HR = 1.41, 95% CI 0.88–2.23, *P* = 0.15). Descriptive subgroup analyses are shown in Supplementary Table S6. We performed interaction tests using Firth penalized Cox regression; the interaction *P*-values were 0.95 for age, 0.65 for diabetes, and 0.40 for albumin. These analyses should be considered exploratory.

## Discussion

In this single-center retrospective cohort study of Chinese PD patients, we observed that a longer BMI TTR within the 26–30 kg/m^2^ range during the first 36 months of dialysis was significantly associated with lower risk of all-cause mortality. For every 10% increment in BMI TTR, the risk of all-cause mortality decreased by approximately 23%. Notably, the TAR threshold scan identified 26 kg/m^2^ as the nadir of mortality risk, while the survival benefit was predominantly driven by sustained maintenance within the 26–30 kg/m^2^ range. These findings suggest that the traditional BMI target may not be optimal for PD patients, but they should be considered exploratory and require confirmation before any clinical recommendations can be made.

It has been widely recognized that BMI is closely associated with mortality in dialysis patients. Over the decades, studies on the relationship between BMI and mortality in PD patients have mainly focused on baseline single-time-point measurements (20), which could only partly reflect nutritional status at a certain time point but could not capture the dynamic weight changes over time. Even though some studies had used time-averaged BMI values or BMI changes between two time points to evaluate this association (21, 22), the detailed dynamic changes and the cumulative exposure to sustained BMI levels might still be overlooked. In fact, the key metric reflecting the dynamic management of body weight is time in range (19), an intuitive metric that denotes the proportion of time during which a patient's BMI is maintained within a specified target range. By introducing this novel metric and using a landmark design to ensure temporal sequence, our study provides, for the first time, evidence that the cumulative duration of sustained BMI exposure above 26 kg/m^2^ is a key driver of survival benefit in PD patients.

Existing studies have not directly evaluated the BMI TTR-mortality association in PD patients; instead, they have relied on baseline BMI or simple BMI changes, which have yielded inconsistent thresholds and failed to provide a quantifiable target for long-term weight management. The absence of such evidence may be due to the traditional reliance on single-time-point measurements in retrospective cohort studies, which do not reflect the cumulative nature of BMI exposure. In contrast, our TAR-TTR framework addresses this gap by integrating all available longitudinal BMI measurements to calculate the cumulative duration of exposure. Furthermore, compared with previous PD cohorts that predominantly included elderly patients (22, 23), our Chinese PD cohort had a younger age distribution, longer follow-up, and more frequent BMI measurements, which may have enhanced our ability to detect the association. The significant inverse association between TTR of 26–30 kg/m^2^ and mortality underscores the clinical relevance of sustained weight management in this population.

In our study, the TAR threshold scan identified 26 kg/m^2^ as the point of lowest mortality risk, and the subsequent TTR analysis showed that the survival benefit was driven by sustained BMI maintenance within 26–30 kg/m^2^. This aligns with Quero et al.'s observation that obesity was not associated with increased mortality in PD patients (23). When a more stringent target range (24–25.9 kg/m^2^) was applied, the association was attenuated and no longer significant, suggesting that the 26–30 kg/m^2^ range may represent a balance between nutritional reserve and metabolic safety.

Several hypothetical mechanisms may explain the association between sustained BMI within 26–30 kg/m^2^ and lower mortality in PD patients. First, a higher BMI may reflect greater nutritional reserve and muscle mass, which could protect against protein-energy wasting (PEW), a common and lethal complication of PD (24). Second, adipose tissue is metabolically active and secretes adipokines that may modulate chronic inflammation and endotoxin load, potentially reducing infection-related mortality (25). Another possible mechanism involves fluid status: BMI can be inflated by overhydration, and the “obesity paradox” in dialysis populations may partly reflect fluid excess rather than true adiposity. Although we could not directly measure overhydration or body composition in this cohort, this does not exclude the possibility that a moderately higher BMI may still reflect better nutritional reserve and muscle mass.

In exploratory competing-risks analyses, the association between BMI-TTR and mortality appeared to differ by cause of death. The potential cardiovascular benefit may be explained by better nutritional reserve, muscle mass, and metabolic stability, which could reduce cardiovascular risk in PD patients (26). In contrast, infection-related mortality is influenced by multiple factors beyond BMI, such as peritonitis episodes, residual kidney function, and dialysis technique (27, 28), and our analysis was underpowered to detect a modest association. These findings suggest that the overall survival benefit associated with BMI-TTR may be largely driven by cardiovascular deaths. Furthermore, our findings suggest that the optimal BMI range for Chinese PD patients may be higher than the Asia-Pacific WHO cut-points for the general population (overweight ≥23 kg/m^2^, obesity ≥25 kg/m^2^) (29), consistent with previous studies reporting better survival in dialysis patients with BMI 25–30 kg/m^2^ or even 28–31 kg/m^2^ (10, 30). However, these mechanisms remain speculative and were not directly examined in this cohort; they require confirmation in future studies with comprehensive nutritional, inflammatory, and body composition assessments, and our findings should not be translated into clinical recommendations without further validation.

Our study has several limitations. First, this single-center retrospective cohort study cannot establish causality and had limited events, yielding an EPV of 10.7 and a *post-hoc* power of approximately 63%. Although Firth penalized Cox regression and step-function TTR sensitivity analyses were consistent, the small number of events may have limited statistical power, particularly for subgroup analyses, and the findings may not be generalizable. Second, residual confounding and measurement error cannot be excluded. BMI cannot distinguish fluid, fat, and lean mass; although we adjusted for 24-h urine volume as a partial proxy, bioimpedance and body composition data were unavailable. PET data were missing in approximately 20% of patients and were not included in the primary model because of EPV limitations, so residual confounding by peritoneal transport status and potential selection bias due to missingness cannot be excluded. While E-values suggested robustness to unmeasured confounding, data on lifestyle factors (e.g., diet, physical activity), Kt/V, and concomitant medications were lacking. In addition, BMI was measured at only three time points, and more frequent measurements could improve the precision of TTR estimation. Third, the 36-month landmark design excluded patients who died or transferred before the landmark. These excluded patients were systematically sicker, which may introduce survivor bias and limit generalizability to patients who survive and remain on PD for at least 36 months. Future studies with more frequent BMI measurements and larger sample sizes are needed to perform competing-risks analyses. Finaly, our cohort was derived from a prefecture-level city in central China, and the findings may not be generalizable to other Chinese regions or Western populations with different ethnic backgrounds. Multi-center studies across diverse populations are needed to confirm our results.

## Conclusion

In summary, sustained maintenance of BMI within the 26–30 kg/m^2^ range was associated with lower all-cause mortality risk in Chinese PD patients. However, given the limited number of events, potential residual confounding by fluid status and body composition, and the single-center design, these findings should be considered exploratory and hypothesis-generating. Further multi-center studies with larger sample sizes and body composition data are needed to confirm whether maintaining BMI above 26 kg/m^2^ can improve clinical outcomes in PD patients.

## Data Availability

The raw data supporting the conclusions of this article will be made available by the authors, without undue reservation.
