# Supplementary material for: The obesity paradox in peritoneal dialysis: reassessing optimal BMI via longitudinal time in range analysis in a single-center cohort
Source: Front Nutr. 2026 Oct 22;13:1982039. doi: 10.3389/fnut.2026.1982039 (PMC13735635; doi:10.3389/fnut.2026.1982039)
Supplement: Supplementary file 1 [file Table_1.doc]

**The obesity paradox in peritoneal dialysis: reassessing optimal BMI via longitudinal time in range analysis in a single-center cohort**

Meihua Wang^1†^, Xiaohong Zeng^2†^, Dehui Liu^1^, Pingli Chen^1*^, Zhiqiang Peng^1*^

**Supplementary material content**

**Table S1.** Baseline characteristics of included and excluded participants.

**Table S2.** Baseline clinical characteristics according to categories of outcome.

**Table S3.** Proportion of missing data for covariates.

**Table S4.** Sensitivity analysis for the association between BMI-TTR of 26-30 kg/m² and death.

**Table S5.** Association between BMI-TTR of 26-30 kg/m² and cause-specific mortality.

**Table S6.** Subgroup descriptive analysis and exploratory interaction tests for the association between BMI-TTR of 26-30 kg/m² and all-cause mortality.

**Figure S1.** Density plots of observed and imputed values for key covariates.

**Figure S2.** The formulas for calculating BMI metrics. Related to Figure 2, Figure 3, Figure 4 and Methods.

**Figure S3.** General study design. Timeline of BMI-TTR exposure and follow-up for clinical outcomes.

**Figure S4.** Distribution of Causes of Death.

Table S1. Baseline characteristics of included and excluded participants.

| **Characteristics** | **Excluded** | **Included** | **p** | **SMD** |
| --- | --- | --- | --- | --- |
|  | 550 | 670 |  |  |
| **Demographic** |  |  |  |  |
| Age, mean (SD), yr. | 52.3 (13.9) | 48.2 (12.5) | <0.001 | 0.312 |
| Male, n (%) | 326 (59.5) | 366 (54.5) | 0.088 | 0.102 |
| **Smoking, n (%)** |  |  | 0.352 | 0.058 |
| no | 466 (85.0) | 585 (87.1) |  |  |
| yes | 82 (15.0) | 87 (12.9) |  |  |
| **Drinking, n (%)** |  |  | 0.066 | 0.111 |
| no | 487 (88.9) | 619 (92.1) |  |  |
| yes | 61 (11.1) | 53 (7.9) |  |  |
| **PET, n (%)** |  |  |  |  |
| Low transport | 26 (5.7) | 41 (6.2) | 0.461 | 0.097 |
| Low average transport | 173 (38.1) | 270 (40.8) |  |  |
| High average transport | 203 (44.7) | 293 (44.3) |  |  |
| High transport | 52 (11.5) | 58 (8.8) |  |  |
| Dialysis month,mean (SD), mon. | 22.05 (15.17) | 58.77 (24.92) | <0.001 | 1.78 |
| **Laboratory test indicators, mean (SD)** |  |  |  |  |
| eGFR (ml/min/1.73m^2^) | 6.62 (3.10) | 6.19 (2.75) | 0.011 | 0.147 |
| UA (umol/L) | 500.75 (159.47) | 520.01 (150.13) | 0.031 | 0.124 |
| CRP (mg/L) | 16.13 (25.84) | 14.12 (23.90) | 0.203 | 0.081 |
| K (mmol/L) | 4.67 (0.89) | 4.62 (0.81) | 0.375 | 0.051 |
| P (mmol/L) | 1.89 (0.66) | 1.89 (0.59) | 0.925 | 0.005 |
| Ca (mmol/L) | 2.02 (0.27) | 2.02 (0.27) | 0.879 | 0.009 |
| iPTH (pg/L) | 369.89 (322.66) | 409.47 (337.98) | 0.04 | 0.12 |
| Ferroprotein (ng/mL) | 287.73 (294.81) | 250.85 (243.91) | 0.019 | 0.136 |
| ALT (U/L) | 19.95 (19.30) | 19.44 (17.64) | 0.628 | 0.028 |
| AST (U/L) | 22.34 (17.33) | 20.94 (13.73) | 0.116 | 0.09 |
| ALB (g/L) | 34.82 (5.60) | 36.23 (5.17) | <0.001 | 0.261 |
| Tcho (mmol/L) | 4.26 (1.18) | 4.34 (1.23) | 0.224 | 0.071 |
| Ldl -c (mmol/L) | 2.48 (0.87) | 2.49 (0.93) | 0.825 | 0.013 |
| TG (mmol/L) | 1.59 (0.87) | 1.60 (0.92) | 0.912 | 0.007 |
| Hdl-c (mmol/L) | 1.16 (0.46) | 1.18 (0.49) | 0.402 | 0.049 |
| Glu (mmol/L) | 5.91 (2.66) | 6.00 (9.71) | 0.85 | 0.012 |
| HGB (g/L) | 81.06 (19.99) | 82.05 (19.29) | 0.379 | 0.051 |
| **Comorbidities,n(%)** |  |  |  |  |
| Hypertension | 496 (90.5) | 611 (90.9) | 0.883 | 0.014 |
| Diabetes | 118 (21.5) | 68 (10.1) | <0.001 | 0.317 |
| Cumulative peritonitis episodes (0-36 month exposure period) | 0.42 (1.00) | 0.48 (0.80) | 0.541 | 0.069 |
| **Examination, mean (SD)** |  |  |  |  |
| 24-hour urine volume (mL) | 879.93 (478.29) | 958.26 (421.49) | 0.002 | 0.174 |
| SBP (mmHg) | 138 (19) | 135 (17) | 0.033 | 0.123 |
| DBP (mmHg) | 84 (11) | 84 (11) | 0.749 | 0.018 |
| BMI (kg/m^2^) | 22.03 (3.33) | 21.90 (3.23) | 0.498 | 0.039 |

Abbreviations: PET,peritoneal equilibration test; eGFR, estimated glomerular filtration rate; UA, uric acid; CRP, C-reactive protein; K,potassium; P,phosphorus; Ca,calcium; iPTH,intact parathyroid hormone; ALB,albumin; ALT,alanine aminotransferase; AST,aspartate aminotransferase; LDL-c, low-density lipoprotein cholesterol; Tcho, total cholesterol; HDL-c, high-density lipoprotein cholesterol; TG, triglyceride; Glu, glucose; HGB, hemoglobin; SBP, systolic blood pressure. DBP, diastolic blood pressure; BMI, body mass index.

**Table S2.** Baseline clinical characteristics according to categories of outcome.

| **Characteristics** | **Overall, n (%)** | **Death, %** | | **p-values** |
| --- | --- | --- | --- | --- |
|  |  | **no** | **yes** |  |
| **Total** | 670 | 595 | 75 |  |
| **Demographic** |  |  |  |  |
| Age, mean (SD), yr. | 48.2 (12.3) | 47.6 (11.9) | 52.9 (13.7) | <0.001 |
| Male, n (%) | 365 (54.5) | 323 (54.3) | 42 (56.0) | 0.875 |
| **Smoking, n (%)** |  |  |  | 0.652 |
| no | 583 (87.0) | 516 (86.7) | 67 (89.3) |  |
| yes | 87 (13.0) | 79 (13.3) | 8 (10.7) |  |
| **Drinking, n (%)** |  |  |  | 1 |
| no | 617 (92.1) | 548 (92.1) | 69 (92.0) |  |
| yes | 53 (7.9) | 47 (7.9) | 6 (8.0) |  |
| **PET, n (%)** |  |  |  |  |
| Low transport | 41 (6.2) | 35 (6.0) | 6 (8.2) | 0.251 |
| Low average transport | 270 (40.9) | 246 (41.9) | 24 (32.9) |  |
| High average transport | 291 (44.1) | 258 (44.0) | 33 (45.2) |  |
| High transport | 58 (8.8) | 48 (8.2) | 10 (13.7) |  |
| Dialysis month, mean (SD), mon. | 58.91 (24.82) | 58.23 (24.98) | 64.25 (23.01) | 0.048 |
| **Laboratory test indicators,**  **mean (SD)** |  |  |  |  |
|  |  |  |  |  |
| eGFR (ml/min/1.73m^2^) | 6.19 (2.75) | 6.17 (2.68) | 6.39 (3.26) | 0.507 |
| UA (umol/L) | 519.28 (148.99) | 520.26 (151.52) | 511.56 (127.78) | 0.634 |
| CRP (mg/L) | 14.16 (23.94) | 14.14 (24.79) | 14.25 (15.77) | 0.972 |
| K (mmol/L) | 4.62 (0.81) | 4.63 (0.81) | 4.55 (0.76) | 0.41 |
| P (mmol/L) | 1.89 (0.59) | 1.90 (0.58) | 1.83 (0.64) | 0.378 |
| Ca (mmol/L) | 3.60 (40.91) | 3.80 (43.41) | 1.99 (0.31) | 0.719 |
| iPTH (pg/L) | 409.89 (338.39) | 412.16 (321.66) | 392.01 (451.21) | 0.628 |
| Ferroprotein (ng/mL) | 251.09 (244.21) | 244.78 (242.42) | 301.11 (254.16) | 0.061 |
| ALT (U/L) | 19.45 (17.67) | 19.51 (18.22) | 18.92 (12.55) | 0.784 |
| AST (U/L) | 20.97 (13.73) | 20.88 (14.16) | 21.71 (9.71) | 0.623 |
| ALB (g/L) | 36.23 (5.16) | 36.34 (5.19) | 35.33 (4.92) | 0.109 |
| Tcho (mmol/L) | 4.34 (1.23) | 4.31 (1.20) | 4.60 (1.46) | 0.056 |
| Ldl -c (mmol/L) | 2.49 (0.93) | 2.49 (0.91) | 2.51 (1.06) | 0.887 |
| TG (mmol/L) | 1.60 (0.93) | 1.60 (0.93) | 1.63 (0.93) | 0.79 |
| Hdl-c (mmol/L) | 1.18 (0.49) | 1.19 (0.50) | 1.13 (0.38) | 0.342 |
| Glu (mmol/L) | 6.00 (9.73) | 6.07 (10.31) | 5.40 (1.58) | 0.576 |
| HGB (g/L) | 82.02 (19.25) | 81.87 (19.41) | 83.25 (18.06) | 0.558 |
| **Comorbidities,n(%)** |  |  |  |  |
| Hypertension | 610 (91.0) | 537 (90.3) | 73 (97.3) | 0.07 |
| Diabetes | 68 (10.1) | 59 (9.9) | 9 (12.0) | 0.719 |
| Cumulative peritonitis episodes (0-36 month exposure period) | 0.48 (0.80) | 0.46 (0.78) | 0.61 (0.92) | 0.136 |
| **Examination, mean (SD)** |  |  |  |  |
| 24-hour urine volume (mL) | 959.03 (421.56) | 963.03 (430.33) | 927.33 (344.97) | 0.49 |
| SBP (mmHg) | 135 (17) | 136 (17) | 131 (14) | 0.014 |
| DBP (mmHg) | 84 (11) | 84 (11) | 81 (9) | 0.016 |
| BMI (kg/m2) | 21.91 (3.24) | 22.00 (3.26) | 21.15 (3.00) | 0.031 |

Abbreviations: PET,peritoneal equilibration test; eGFR, estimated glomerular filtration rate; UA, uric acid; CRP, C-reactive protein; K,potassium; P,phosphorus; Ca,calcium; iPTH,intact parathyroid hormone; ALB,albumin; ALT,alanine aminotransferase; AST,aspartate aminotransferase; LDL-c, low-density lipoprotein cholesterol; Tcho, total cholesterol; HDL-c, high-density lipoprotein cholesterol; TG, triglyceride; Glu, glucose; HGB, hemoglobin; SBP, systolic blood pressure. DBP, diastolic blood pressure; BMI, body mass index.

Table S3. Proportion of missing data for covariates.

| **Variant** | **Proportion (%)** |
| --- | --- |
| Age | 0 |
| Smoking | 0 |
| Drinking | 0 |
| Diabetes | 0 |
| Hypertension | 0 |
| eGFR | 0 |
| ALB | 0 |
| 24-hour urine volume | 0 |
| SBP | 0 |
| DBP | 0 |
| BMI | 0 |
| K | 0 |
| P | 0 |
| Ca | 0 |
| ALT | 0 |
| AST | 0 |
| UA | 0 |
| HGB | 0 |
| iPTH | 0.7 |
| TG | 1.04 |
| LDL-c | 1.2 |
| Tcho | 1.2 |
| ferritin protein | 1.3 |
| HDL-c | 1.3 |
| PET | 1.5 |
| Glu | 1.8 |
| CRP | 15.9 |
| Cumulative peritonitis episodes (0-36 month exposure period) | 15.9 |

Abbreviations: PET,peritoneal equilibration test; eGFR, estimated glomerular filtration rate; UA, uric acid; CRP, C-reactive protein; K,potassium; P,phosphorus; Ca,calcium; iPTH,intact parathyroid hormone; ALB,albumin; ALT,alanine aminotransferase; AST,aspartate aminotransferase; LDL-c, low-density lipoprotein cholesterol; Tcho, total cholesterol; HDL-c, high-density lipoprotein cholesterol; TG, triglyceride; Glu, glucose; HGB, hemoglobin; SBP, systolic blood pressure. DBP, diastolic blood pressure; BMI, body mass index.

**Table S4.** Sensitivity analysis for the association between BMI-TTR of 26-30 kg/m² and death .

| **Variables** | **cases/person years** | **Model 1** | | **Model 2** | | **Model 3** | |
| --- | --- | --- | --- | --- | --- | --- | --- |
|  |  | HR (95% CI) | P value | HR (95% CI) | P value | HR (95% CI) | P value |
| **Using the complete dataset without imputation (N=554)** | | | | | | | |
| **as category variable** |  |  |  |  |  |  |  |
| Never within target (TTR = 0%) | 69/1429.66 | Reference |  | reference |  | reference |  |
| Any time within target (TTR > 0%) | 5/314.69 | 0.33(0.12-0.82) | 0.01 | 0.30(0.12-0.76) | 0.01 | 0.27(0.11-0.70) | 0.006 |
| **as continuous variable** |  |  |  |  |  |  |  |
| BMI-TTR of 26-30kg/m^2^ per 10% increment |  | 0.80(0.65-0.99) | 0.04 | 0.79(0.64-0.97) | 0.03 | 0.78(0.63-0.97) | 0.02 |
| **Using Step-function method to calculate TTR (N=670)** | | | | | | | |
| **as category variable** |  |  |  |  |  |  |  |
| Never within target (TTR = 0%) | 69/1497.49 | Reference |  | reference |  | reference |  |
| Any time within target (TTR > 0%) | 6/361.84 | 0.36(0.15-0.83) | 0.02 | 0.32(0.14-0.74) | 0.01 | 0.29(0.13-0.70) | 0.007 |
| **as continuous variable** |  |  |  |  |  |  |  |
| BMI-TTRof 26-30kg/m^2^ per 10% increment |  | 0.87(0.78-0.98) | 0.03 | 0.86(0.76-0.97) | 0.015 | 0.85(0.75-0.96) | 0.012 |
| **Firth penalized Cox regression (N=670)** | | | | | | | |
| **as category variable** |  |  |  |  |  |  |  |
| Never within target (TTR = 0%) | 70/1524.00 | Reference |  | reference |  | reference |  |
| Any time within target (TTR > 0%) | 5/335.34 | 0.36(0.13-0.77) | 0.006 | 0.32(0.12-0.71) | 0.003 | 0.29(0.12-0.65) | 0.001 |
| **as continuous variable** |  |  |  |  |  |  |  |
| BMI-TTR of 26-30kg/m^2^ per 10% increment |  | 0.82(0.65-0.96) | 0.01 | 0.81(0.64-0.95) | 0.005 | 0.80 (0.63-0.94) | 0.004 |

Notes:

Model 1: crude model.

Model 2: adjusted for age, diabetes.

Model 3: adjusted for adjusted for age, diabetes, eGFR, ALB, HDL-c, 24-hour urine volume, cumulative peritonitis episodes during the 0-36 month exposure period.

**Table S5.** Association between BMI-TTR of 26-30 kg/m² and cause-specific mortality.

| **Variables** | **cases/person years** | **Model 1** | | **Model 2** | | **Model 3** | |
| --- | --- | --- | --- | --- | --- | --- | --- |
|  |  | HR (95% CI) | P value | HR (95% CI) | P value | HR (95% CI) | P value |
| **cardiovascular-related mortality (N=670)** | | | | | | | |
| **as category variable** |  |  |  |  |  |  |  |
| Never within target (TTR = 0%) | 35/1524.00 | Reference |  | reference |  | reference |  |
| Any time within target (TTR > 0%) | 3/335.34 | 0.40(0.12-1.31) | 0.13 | 0.40(0.13-1.22) | 0.11 | 0.38(0.13-1.14) | 0.08 |
| **as continuous variable** |  |  |  |  |  |  |  |
| BMI-TTR of 26-30kg/m^2^ per 10% increment |  | 0.69(0.52-0.91) | 0.01 | 0.69(0.53-0.89) | 0.004 | 0.68(0.53-0.88) | 0.004 |
| **Infection-related mortality (N=670)** | | | | | | | |
| **as category variable** |  |  |  |  |  |  |  |
| Never within target (TTR = 0%) | 19/1524.00 | Reference |  | reference |  | reference |  |
| Any time within target (TTR > 0%) | 2/335.34 | 0.52(0.12-2.23) | 0.38 | 0.39(0.07-2.10) | 0.27 | 0.37(0.07–2.04) | 0.26 |
| **as continuous variable** |  |  |  |  |  |  |  |
| BMI-TTR of 26-30kg/m^2^ per 10% increment |  | 0.96(0.75-1.24) | 0.78 | 0.93(0.70-1.23) | 0.61 | 0.93 (0.71–1.21) | 0.58 |

Notes:

Model 1: crude model.

Model 2: adjusted for age, diabetes.

Model 3: adjusted for adjusted for age, diabetes, eGFR, ALB, HDL-c, 24-hour urine volume, cumulative peritonitis episodes during the 0-36 month exposure period.

**Table S6.** Subgroup descriptive analysis and exploratory interaction tests for the association between BMI-TTR of 26-30 kg/m² and all-cause mortality.

| **Subgroup** | **Level** | **No. of patients** | **Deaths** | **mortality rate** | **Interaction Term** | **P for interaction** |
| --- | --- | --- | --- | --- | --- | --- |
| **Age** | <65 | 616 | 60 | 9.7 | BMI-TTR of 26-30 kg/m² per 10% increment:Age | 0.95 |
|  | ≥65 | 54 | 15 | 27.8 |  |  |
| **Diabetes** | No | 602 | 66 | 11 | BMI-TTR of 26-30 kg/m² per 10% increment:Diabetes | 0.65 |
|  | Yes | 68 | 9 | 13.2 |  |  |
| **Albumin** | Low | 336 | 38 | 11.3 | BMI-TTR of 26-30 kg/m² per 10% increment:Alb | 0.40 |
|  | High | 334 | 37 | 11.1 |  |  |

**Figure S1.** Density plots of observed and imputed values for key covariates.

Notes:

The blue lines represent the observed data, and the red lines represent the imputed values from five imputed datasets. The imputed distributions closely mirror the observed distributions, supporting the validity of the multiple imputation model under the missing-at-random assumption.

**Figure S2.** The formulas for calculating BMI metrics. Related to Figure 2, Figure 3, Figure 4 and Methods.

**Figure S3.** General study design. Timeline of BMI-TTR exposure and follow-up for clinical outcomes.

Abbreviations: BMI, body mass index; TTR, time in target range.

**Figure S4.** Distribution of Causes of Death.
